# IGFBP5, as a Prognostic Indicator Promotes Tumor Progression and Correlates with Immune Microenvironment in Glioma

**DOI:** 10.7150/jca.87733

**Published:** 2024-01-01

**Authors:** Jiediao Lin, Guowei Huang, Qianru Zeng, Rendong Zhang, Yun Lin, Yaochen Li, Baohua Huang, Hongchao Pan

**Affiliations:** 1 Central laboratory, Cancer Hospital of Shantou University Medical College, Shantou, Guangdong 515041, China.; 2 Guangdong Provincial Key Laboratory for Breast Cancer Diagnosis and Treatment, Cancer Hospital of Shantou University Medical College, Shantou, Guangdong 515041, China.; 3 Department of Pathology, Shantou University Medical College, Shantou, Guangdong, 515041, China.; 4 The Breast Center, Surgical Oncology Session No. 1, Cancer Hospital of Shantou University Medical College, Shantou, Guangdong 515041, China.; 5 Department of Pathology, Shantou Central Hospital, Shantou, Guangdong 515041, China.

**Keywords:** Glioma, IGFBP5, Invasion, Immune infiltration, Prognostic marker

## Abstract

**Background:** Insulin-like growth factor binding protein 5 (IGFBP5) is highly expressed in multiple human cancers, including glioma. Despite this, it remains unclear what role it plays in glioma. The aim of the present study was to analyze whether IGFBP5 could be used as a predictor of prognosis and immune infiltration in glioma.

**Methods:** Glioma patients' clinical information was collected from the Cancer Genome Atlas (TCGA), the Chinese Glioma Genome Atlas (CGGA), Rembrandt, and Gravendeel databases. The diagnostic and prognostic roles of IGFBP5 were assessed by the Kaplan-Meier survival curves, diagnostic receiver operating characteristic (ROC) curves, nomogram model, Cox regression analysis and Enrichment analysis by R software. Moreover, the correlation between IGFBP5 expression and immune cell infiltration, and immune checkpoint genes was conducted. Immunohistochemistry staining, CCK8, colony formation, scratch and transwell assays and western blot were used to interrogate the expression and function of IGFBP5 in glioma.

**Results:** IGFBP5 levels were obviously increased in glioma with higher malignancy and predicted poor outcomes by Univariate and multivariate Cox analysis. The biological function analysis revealed that IGFBP5 correlated closely with immune signatures. Moreover, IGFBP5 expression was associated with tumor infiltration of B cells, T cells, macrophages, and NK cells. IGFBP5 affected glioma cell proliferation, migration, and invasion probably involved in the epithelial-to-mesenchymal transition (EMT) and Hippo-YAP signaling pathway. Further study showed that IGFBP5 induced the expression of PD-L1 and CXCR4.

**Conclusions:** IGFBP5 as an oncogene is a useful biomarker of prognosis and correlates with progression and immune infiltration in glioma.

## Introduction

Glioma, including lower grade glioma (LGG) and glioblastoma multiforme (GBM), accounts for 81% of intracranial malignancies 1. Glioma have been classified into four grades: I, II, III, and IV, in which glioma of Grades I and II are called low grade gliomas (LGG), while glioma of Grades III and IV are called high grade gliomas (HGG) 2. Glioma patients have a poor prognosis, and the median survival time is only 12-18 months 3. Glioma remains a stubbornly low-survival disease despite multiple treatment options, including surgery, chemotherapy, radiotherapy, and immunotherapy. Therefore, it is urgent need to identify reliable, prognostic biomarkers and reveal the molecular mechanisms for glioma treatment.

IGF binding proteins (IGFBPs) are a family of six structurally homologous members, which bind and regulate the effects of IGFs on the target cells, including cell growth, migration, and invasion 4. The expression of IGFBP5, the most conserved of the IGFBPs, is aberrant in tumors 5. The IGFBP5 can bind to domain of conserved amino acid domains in IGF1 and influence the IGF1 receptor (IGF1R) binding to regulate IGFI-dependent and IGFI-independent cellular functions 6. Despite increasing evidences have shown that IGFBP5 expression has been correlated with cancer development, its accurate role in different cancers is controversial 7. Some research has shown that IGFBP5 was implicated in numerous malignancies as an oncogene 8, while other studies have shown that IGFBP5 could restrain tumor growth and metastasis 9. Few functional and immune insights into IGFBP5 in glioma have been provided in previous studies. Wang et al. reported that IGFBP5 expression was significantly correlated with glioma histologic grade, which indicated that IGFBP5 might play a role in glioma progression 10. Dong et al. showed that IGFBP5 induced cell invasion, but suppressed cell proliferation 11. Nonetheless, the distinct roles of IGFBP5 in glioma, particularly in relation to molecular mechanisms, tumor microenvironment and immune status, remain unclear.

Here, our study indicates the prognostic implications of IGFBP5 in glioma by data mining of datasets from CGGA and TCGA, Rembrandt and Gravendeel databases. Subsequently, our results show that IGFBP5 expression has positively correlated with the infiltration of immune cells in glioma, including TAM and M2 macrophages. Moreover, our study further demonstrates IGFBP5 participates in proliferation and invasion involved in EMT and Hippo-YAP signaling pathway in glioma cells, and influences the expression of PD-L1 and CXCR4 expression. These results reveal that IGFBP5 is correlated with immune cell infiltration and exert a critical role in the development of glioma, and may serve as a prognostic biomarker in glioma patients.

## Materials and methods

### Data collection

We retrieved clinical data from TCGA (comprising 11069 samples from 33 types of cancer) by using UCSC Xena (https://xena.ucsc.edu/) and gene profile data for normal human tissues from GTEx (https://commonfund.nih.gov/GTEx). In this study, all of the glioma expression matrices (both GBM and LGG) were obtained from the TCGA database and three databases (containing CGGA, Rembrandt, and Gravendeel databases) from the gliovis online site (http://gliovis.bioinfo.cnio.es/), including 4 datasets containing 2231 samples: 637 grade II patients, 749 grade III patients and 845 grade IV patients (Supplementary Table 1). UALCAN portal (http://ualcan.path.uab.edu/analysis-prot.html) was used to analyze the Clinical proteomic tumor analysis consortium (CPTAC) data. Using the limma R, all gene expression data were batch processed and normalized. We excluded individuals with unknown or partial data from the analyzed dataset and only kept the glioma with complete clinicopathological data and survival data.

### IGFBP5 differential expression in glioma

Using Wilcoxon rank sum and signed rank tests, we conducted differential IGFBP5 analysis on the pan cancer expression profile data received from UCSC. We examined the expression of IGFBP5 in glioma and normal brain tissuesby the TCGA + GTEx, Rembrandt, and Gravendeel databases. Additionally, we contrasted IGFBP5 expression in glioma with other clinical traits, including age, gender, grade (WHO II, WHO III, and WHO IV), and histological status.

### Relationship between IGFBP5 expression, immune infiltration and immune cell markers in glioma

The data of glioma patients obtained from TCGA database and the link between IGFBP5 expression and immune infiltration were analyzed using CIBERSORT and EPIC algorithms by R software immuneeconv package. To further validate the above analysis, we assessed the relationship between IGFBP5 expression and the tumor infiltration status of 24 immune cell types using the ssGSEA algorithm in the "GSVA" (v1.34.0) R package. The correlation of IGFBP5 and StromalScore, ImmuneScore, ESTIMATEScore was analyzed using Estimate algorithm by R software package ggplot2. The correlation of IGFBP5 and immune cell markers in glioma was analyzed using R software ggplot2 package by TCGA and CGGA databases.

### Correlation between IGFBP5 expression and immunomodulators in glioma

The correlation between the IGFBP5 expression and immunomodulator genes (including immunoinhibitors, immunostimulators and MHC moleculars) was analyzed in glioma samples from the TCGA database using the Spearman's correlation analysis with the “ggplot2” R package. The correlation was deemed significant when the threshold was set at p < 0.05.

### Validation of the prognostic ability of IGFBP5

In order to further study the prognostic value of IGFBP5 in glioma, the forest plot was used to display the P value and risk ratio (HR) with 95% confidence interval (CI) of IGFBP5 through R package “forestplot” (http://www.bioconductot.org). Univariate and multivariate Cox regression analyses were performed by the 'rms'R package to construct the OS nomogram model. Nomograms are quantitative analysis plots that represent a functional relationship between multiple variables with a cluster of mutually disjoint line segments in planar coordinates. Clinical characteristics such as age, gender, grade, IDH molecular characteristics, 1p19q, Primary therapy outcome, and IGFBP5 expression were used to construct a nomogram. The OS probability of patients with glioma for 1-, 3-, and 5 -year was determined by use of the rms R package.

### Function and pathway enrichment analysis

Based on the median IGFBP5 expression levels in TCGA cohort, differentially expressed genes (DEGs) from glioma in high-and low-expressed groups were identified by limma package on R with the criteria P < 0.05, |logFC0| > 1.5. The Gene Ontology (GO) function annotation analysis and Kyoto Encyclopedia of Genes and Genomes (KEGG) pathway enrichment analysis were carried out for common DEGs using the R software package clusterProfiler.

### Cell culture and tissue samples

Glioma tissue chips purchased from US Biomax Inc, MD, USA (Cat. #BS17015b and #GL242a) and tested in parallel. The samples from both microarrays contained 9 normal brain tissues and 76 pathological sections of glioma patients, including 50 cases of astrocytoma, 6 cases of Oligodendroglioma and 20 cases of glioblastoma from 46 male and 30 female patients. Human normal glial cell HEB, and glioma cells including SHG44, T98, U251 and U87 were bought from the Chinese Type Culture Collection (CTCC, Shanghai, China) and cultured in DMEM (Cat. #SH30021.01, Hyclone) supplemented with 10% fetal bovine serum (Cat. #11011-8611, Sijiqing, Hangzhou, China), and 1% penicillin-streptomycin at 37°C and 5% CO_2_. These cell lines were confirmed by short tandem repeat (STR) analysis.

### Transduction, RNA extraction and qRT-PCR

The siRNAs for IGFBP5 (NM_000599) were bought from Genefarm company (Suzhou, China). The sequences for the control siRNA (NC) and three sequences for IGFBP5 siRNAs were shown in Supplementary Table 2. The plasmid of pCMV3-IGFBP5-GFP (Cat. #HG10206-ACG) and vector plasmid pCMV3-C-GFP (Cat. #CV026) were purchased from Sino Biological company (Beijing). The siRNA or plasmids were transfectioned with Lipofectamine 3000 (Life Technologies) according to the instructions of the manufacturer. qRT-PCR and Western-blot analyses were used to verify the effect of the IGFBP5 siRNAs. Total RNA was obtained using TRIzol reagent (Life technologies, USA) in accordance with manufacturer's instructions. The primers sequences used for qRT-PCR were shown in Supplementary Table 2. Relative expression values were computed by the 2^-ΔΔCt^ method. Statistical analyses were performed using the GraphPad Prism.

### Immunohistochemistry (IHC)

Immunohistochemistry (IHC) experiments were performed as previously described 12. Briefly, after deparaffinization and rehydration, the tissue sections were boiled in 10 mM sodium citrate buffer (pH 6.0) for 30 min, then cooled under room temperature. After blocking endogenous peroxidase activity with 3% H_2_O_2,_ IGFBP5 antibody (1:500, Cat. #55205-1-AP, Proteintech) incubated overnight at 4ºC. After washing, slides were incubated with immunoreaction enhancer solution (Cat. #PV- 9000, ZSGB-BIO), then washed three times and incubated with horseradish peroxidase (HRP)-conjugated goat anti-rabbit secondary antibody for 1 hours. Sections were then incubated for 5 min in an AEC solution before being stained with hematoxylin. For imaging, each of the slides selected 3 random fields acquired with Zeiss Axio-Imager (Carl Zeiss Microscopy GmbH, Oberkochen, Germany). Immunohistochemical scores were obtained by determining the intensity and density of staining using ImageJ software. Immunohistochemical scores were calculated by evaluating staining intensity and density using ImageJ software. Scores for immunohistochemical staining intensity (ISI) were: 0 = negative; 1 = mild staining; 2 = moderate staining; 3 = strong staining, and scores for immunohistochemical staining density (ISD) were: 0 = no positive cells; 1 = 1-25% of positive cells; 2 = 26-75% of positive cells; 3 ≧ 75% of positive cells. The final histochemical score was based on multiplying the ISI and ISD scores for each condition. A score ≦ 4 was regarded as representing a weak expression, and a score > 4 was considered to represent a high expression.

### Cell proliferation and colony formation assay

To detect cell proliferation, the Cell Counting Kit-8 (CCK-8, E606335-0500, NCM, Suzhou) was employed. U251 and U87 cells were digested and planted in 96-well culture plates with five identical wells serving as duplicate wells at a density of 2×10^3^ cells per well, followed by overnight incubation in a humidified incubator. Then, at different time points of 24 hours, 48 hours, and 72 hours, 10 μL of CCK-8 reagent was added into each well and incubated for 2 hours. Absorbance was measured at 450 nm. The assays were independently performed three times. For colony formation, U251 cells were transfected with IGFBP5 vector or empty vector was cultured by the medium contained 200ug/ml Hydromycin for 2 weeks and selected the cells stably expressed IGFBP5, then 800 cells were seeded on the 6-well plates and cultured for 2 weeks. The colonies were stained with 0.1% crystal violet, then visualized by a phase-contrast light microscopy. Cumulative growth of more than 50 cells was identified as the formation of a colony.

### Wound healing analysis

Cells (1 × 10^5^/100 μL/well) were inoculated in 96-well plate and cultured overnight at 37°C. Then, an artificial wound was created in the monolayer with a 10μL pipette tip and washed with DMEM. After incubation for 24 h, the wound distance was captured using a microscope and analyzed using the ImageJ software.

### Transwell migraion and invasion assays

U251 and U87 cells transfected with si-NC or si-IGFBP5 were seeded at 5 × 10^4^ per well with FBS-free medium onto the upper Transwell chambers (8-μm pore size, Corning), which were not pretreated for migration assay or pretreated with the matrigel coated the chamber for invasion assay incubated at 37 °C for 30 min. And 500 μL DMEM contained 10% FBS was added into the lower chamber. After incubation for 24 h, cells passing through the pores were fixed with 4% paraformaldehyde and stained with crystal violet. And the non-penetrating cells in the upper lumen were erased with a cotton swab. Quantitative analysis of penetrated cells by five visual fields using ImageJ.

### Western blot assay

The protein was extracted by RIPA lysis and equal amounts of protein were separated by SDS-PAGE and transferred onto PVDF membrane. After sealing the membrane with 5% BSA in TBST for 1 hour at room temperature, the primary antibodies were incubated overnight at 4°C, followed by incubation of the corresponding peroxidase-labeled secondary antibody for 1 hour at room temperature. Using the ECL solution (Santa Cruz, Dallas, TX, USA), the protein bands were visualized. GAPDH served as the loading control. The following primary antibodies were used: IGFBP5 (1:500, Cat. #55205-1-AP), PD-L1 (1:1000, Cat. #28076-1-AP) , GAPDH (1:2000, Cat. #60004-1-Ig) were bought from Proteintech (Chicago, USA). Vimentin (1:1000, Cat. #5741), N-cadherin (1:1000, Cat. #13116), E-cadherin (1:1000, Cat. #4691), Kibra (1:1000, Cat. #8774) were purchased from Cell Signaling Technology (Danvers, MA, USA). CXCR4 (1:1000, Cat. #4060) was bought form Sigma-Aldrich (Oslo, Norway, USA). MST1/MST2 (1:1000, Cat. #DF8569) and p-YAP (1:1000, Cat. #AF3328) was bought form Affinity. YAP1 (1:1000, Cat. #ab76252) was bought form Abcam. p-MST1/MST2 (1:1000, Cat. #PA5-40255) was bought form Invitrogen.

### Statistical analysis

Data were expressed as mean ± standard deviation (SD). Comparisons between two groups were analyzed using the Student's t-test. Comparisons among multiple groups were made using one-way analysis of variance (ANOVA) using Prism 9 (GraphPad Software Inc. CA, USA). All experiments were performed at least three times. Differences between samples were considered significant at ^*^ *P* < 0.05, ^**^ P < 0.01, and^ ***^ *P* < 0.001.

## Results

### Differential expression analysis of IGFBP5 in glioma

**Figure 1:**
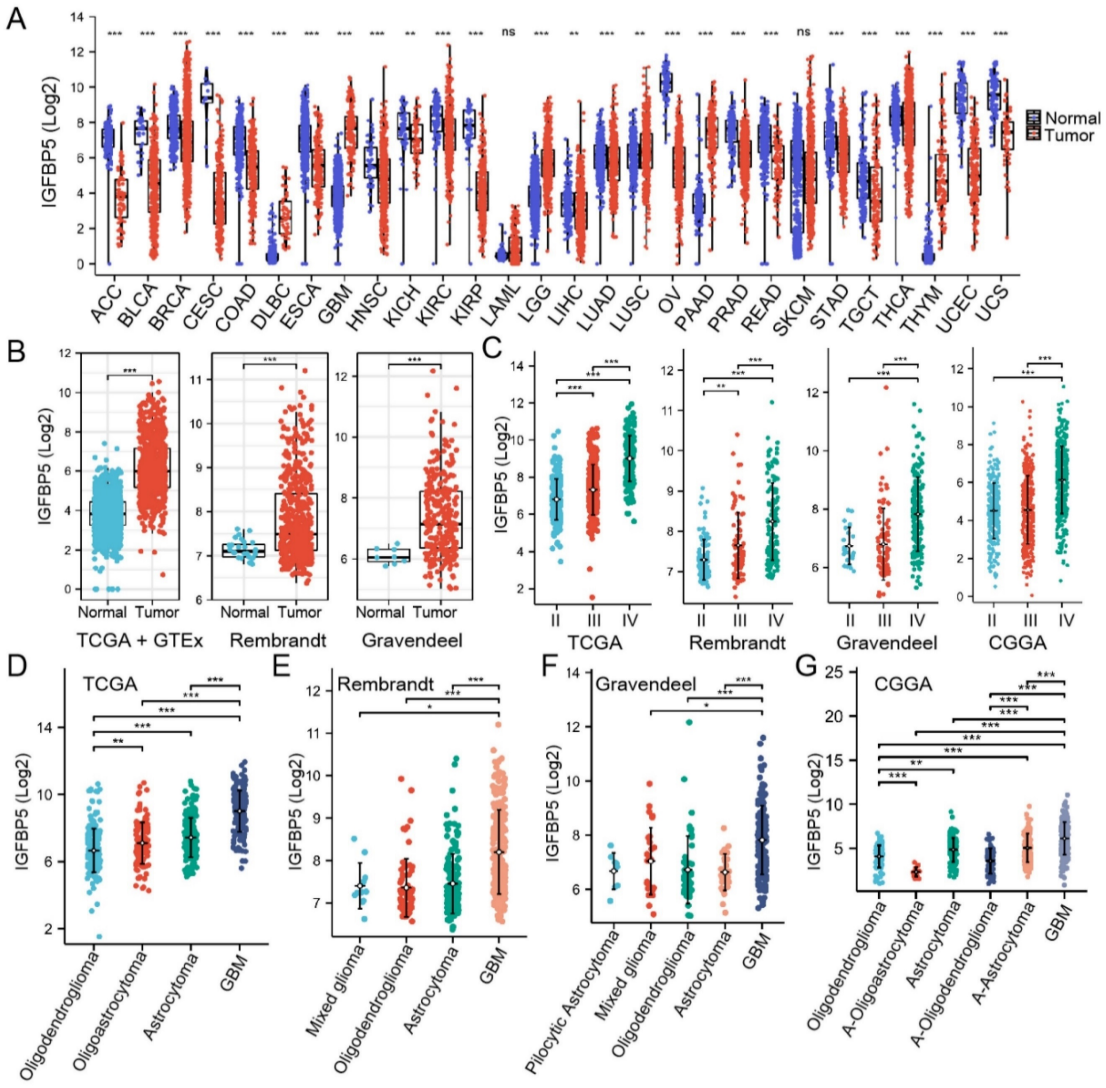
Expression analysis of IGFBP5. **(A)** IGFBP5 mRNA expression in different cancers and normal tissues. ^*^P < 0.05; ^* *^P < 0.01; ^* * *^P < 0.001; ns = no signifcance (Wilcoxon test). **(B)** Differential expression analysis of IGFBP5 between TCGA + GTEx database, Rembrant, and Gravendeel cohorts. **(C)** IGFBP5 expression significantly correlates with WHO grade in TCGA database, Rembrant, Gravendeel and CGGA cohorts. **(D-F)** IGFBP5 expression significantly correlates with sub-type of glioma in TCGA database (D), Rembrant (E), Gravendeel (F), and CGGA (G) cohorts.^ *^P < 0.05; ^* *^P < 0.01; ^* * *^P < 0.001.

We utilized RNA-seq data from TCGA and GTEX database to analyze the expression of IGFBP5 between various tumors and normal tissues, and the results showed the IGFBP5 expression in Lymphoid Neoplasm Diffuse Large B-cell Lymphoma (DLBC), glioblastoma multiforme (GBM), brain lower grade glioma (LGG), lung squamous cell carcinoma (LUSC), pancreatic adenocarcinoma (PAAD), and thymoma (THYM) was higher than that in normal tissues (Figure 1A). In comparison to normal tissues, IGFBP5 expression was significantly reduced in all remaining cancers but not statistically different in LAML and SKCM (Figure 1A). We found that IGFBP5 was significantly elevated in both LGG and GBM, so we subsequently focused on the function of IGFBP5 in glioma. Using data from the TCGA, GTEx, Rembrand and Gravendel datasets, we further found that IGFBP5 expression was upregulated in glioma compared to normal tissues (Figure 1B). To study the correlation between IGFBP5 expression and WHO grade for glioma, we analyzed the data from TCGA, Rembrand, Gravendeel and CGGA cohorts and the results discoursed that IGFBP5 expression was gradually increased responding to tumor grade through II-IV in these four datasets (Figure 1C). We further examined IGFPB5 expression in different histology of glioma in four datasets and the data revealed that GBM had the higher expression level of IGFBP5 than other histology types (Figure 1D-G). Collectively, IGFBP5 expression is upregulated in glioma and gradually increases with the progress of tumor grade. To analyze the correlation between the IGFBP5 expression levels and Clinical parameters, we used the R package to analyze the samples of the glioma cohort TCGA database. High expression of IGFBP5 was significantly correlated with age, WHO grade (62 samples with missing WHO grade information), IDH status (10 samples with missing IDH status information), primary therapy (234 samples with missing primary therapy outcome information) and 1p/19q codeletion (7 samples with missing 1p/19q codeletion information) (Supplementary Figures 1 and Table 1). However, there was no significant relationship between IGFBP5 expression and gender (Supplementary Figures 1 and Table 1). The findings show that elevated IGFBP5 expression increases malignant degree of glioma and results in a worse prognosis for patients, further confirming that IGFBP5 may be an oncogene of glioma.

### IGFBP5 as an indicator for survival prediction

**Figure 2:**
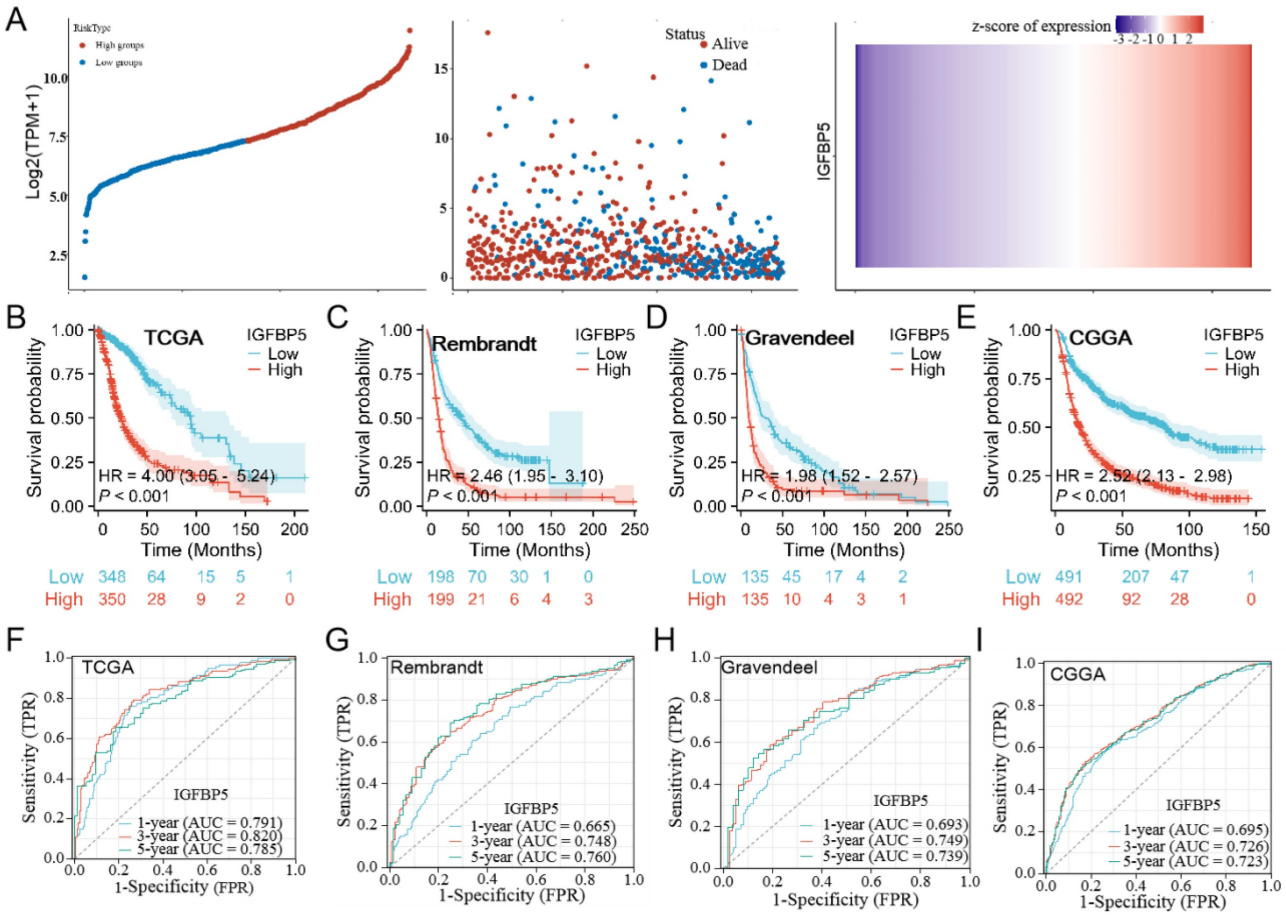
Associations between IGFBP5 expression and prognosis of patients in different datasets. **(A)** Risk model for IGFBP5 in glioma by TCGA database. The top scatterplot represents IGFBP5 expression from low to high (top left). Different colors represent different groups. The scatter plot distribution represents IGFBP5 expression of different samples correspond to the survival time and survival status (middle left). The buttom figure is IGFBP5 mRNA expression heatmap (bottom left). **(B-E)** Kaplan-Meier curves estimated overall survival of IGFBP5 expression for glioma in four databases. **(F-I)** Time-dependent ROC curves of overall survival at 1, 3, and 5 years.The higher values of AUC corresponding to higher predictive power.

To investigate the effect of IGFBP5 on the prognosis of glioma, patients with glioma were divided into low- and high groups according to IGFBP5 expression.The results showed the distribution of risk scores and and the number of deaths were higher in the high-risk score group than in the low-risk score group in the glioma dataset (Figure 2A). Using the TCGA, CGGA, Rembrandt, and Gravendeel datasets, we investigated the predictive potential of IGFBP5 on the prognosis of glioma patients. The Kaplan-Meier curves demonstrated that high expression of IGFBP5 was remarkably associated with poor OS in all the four datasets (Figure 2B-E), suggesting high IGFBP5 expression can be an independent risk factor to predict the prognosis of glioma patients. Assessment of the prognostic value of IGFBP5 expression in glioma patients subgrouped by age (age < 60, age ≥ 60), gender (female, male), IDH mutation status (mutant, wild type), WHO grade (WHO 2, WHO 3 & WHO 4), and primary therapy outcome (PD, SD, PR, and CR) was conducted. The survival analysis revealed that high expression of IGFBP5 was associated with poor prognosis in age, gender, WHO, IDH mutant, PD and SD groups (Suppl Figure 2A-E). However, there was no difference in the IDH wild type, PR and CR groups with expression of IGFBP5 (Suppl Figure 2D, E). In addition, to assess the predictive efficiency of IGFBP5 expression levels on 1-, 3- and 5-year survival in glioma, we performed ROC curves using data from the four datasets mentioned above. In TCGA cohort, the 1-, 3-, and 5-year AUCs were 0.791 (95% CI, 0.745 to 0.837), 0.820 (95% CI, 0.776 to 0.863), and 0.785 (95% CI, 0.727 to 0.843), respectively (Figure 2F). In CGGA cohort, the 1-, 3-, and 5-year AUCs were 0.695 (95% CI, 0.658 to 0.733), 0.726 (95% CI, 0.694 to 0.761), and 0.723 (95% CI, 0.685 to 0.761), respectively (Figure 2G). Similarly, the 1-, 3-, and 5-year AUCs were greater than 0.65 in Rembrandt and Gravendeel cohorts (Figure 2H, I). All the results suggest the appreciable reliability of IGFBP5 as biomarker for glioma prognosis.

**Figure 3:**
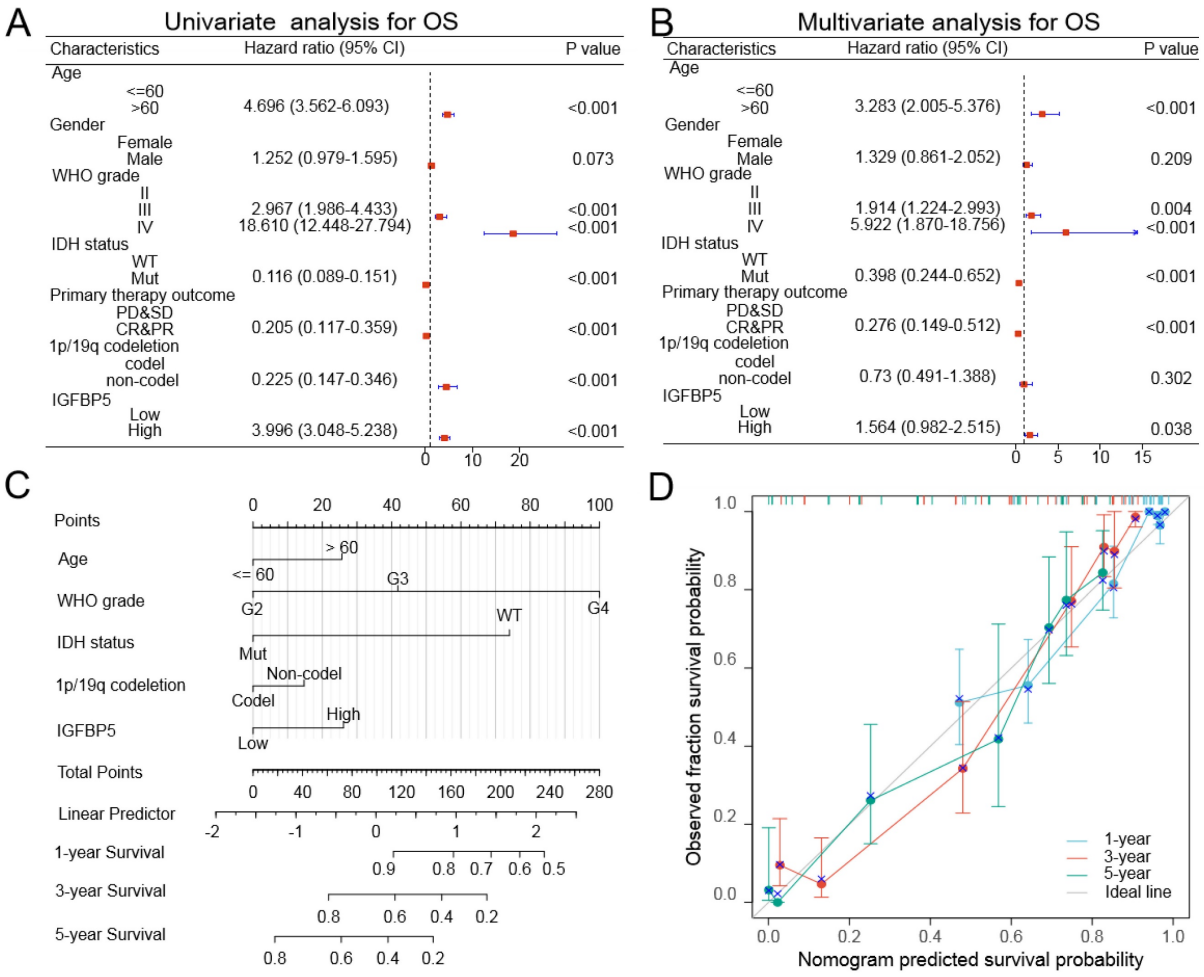
Univariate and multivariate Cox regression analyses regarding OS for IGFBP5 in TCGA dataset. **(A, B)** Unavailable and multivariable analyses of overall survival in glioma patients. The blue squares on the transverse lines represent the hazard ratio (HR), and the dot transverse lines represent 95% CI.** (C)** Nomogram, calibration plots and clinical impact plots for the prediction of OS survival at 1, 3 and 5 y in glioma patients. C-index: 0.845 (0.834-0.856). **(D)** The calibration plots for predicting OS at 1, 3 and 5 y, diagonal line: ideal model; vertical bars: 95% confidence interval.

To further confirm whether IGFBP5 was an independent prognostic factor in glioma patients, we conducted univariable and multivariable Cox regression analysis used the TCGA dataset through 'forestplot' R package. Univariate Cox analysis showed that age, gender, WHO grade, IDH mutation status, Primary therapy outcome, 1p/19q deletion status, and IGFBP5 expression were closely correlated with the prognosis of glioma in TCGA dataset (Figure 3A). Multivariate Cox analysis revealed that age, WHO grade, IDH mutation status, Primary therapy outcome, 1p/19q deletion status, and IGFBP5 were independent prognostic factors in TCGA dataset (Figure 3B). In this study, a nomogram model was established using age, gender, grade, IDH status and IGFBP 5 expression level as parameters of the OS predicting model by using 'rms' R package. Based on a multiple-Cox regression analysis, the factors were identified as highly prognostically predicting factors. The nomogram for predicting survival rates of the 1-, 3- and 5-year group in glioma patients demonstrated significantly higher clinical value (Figure 3C). In the calibration curves for the TCGA cohort (Figure 3D), the predictive curve achieved high accuracy for prediction of the 1-, 3- and 5-year OS of glioma patients (C-index 0.845, 0.834 to 0.856). These results validated that IGFBP5 was an independent prognostic factor of glioma.

### Correlation and enrichment analyses of IGFBP5 in TCGA

**Figure 4:**
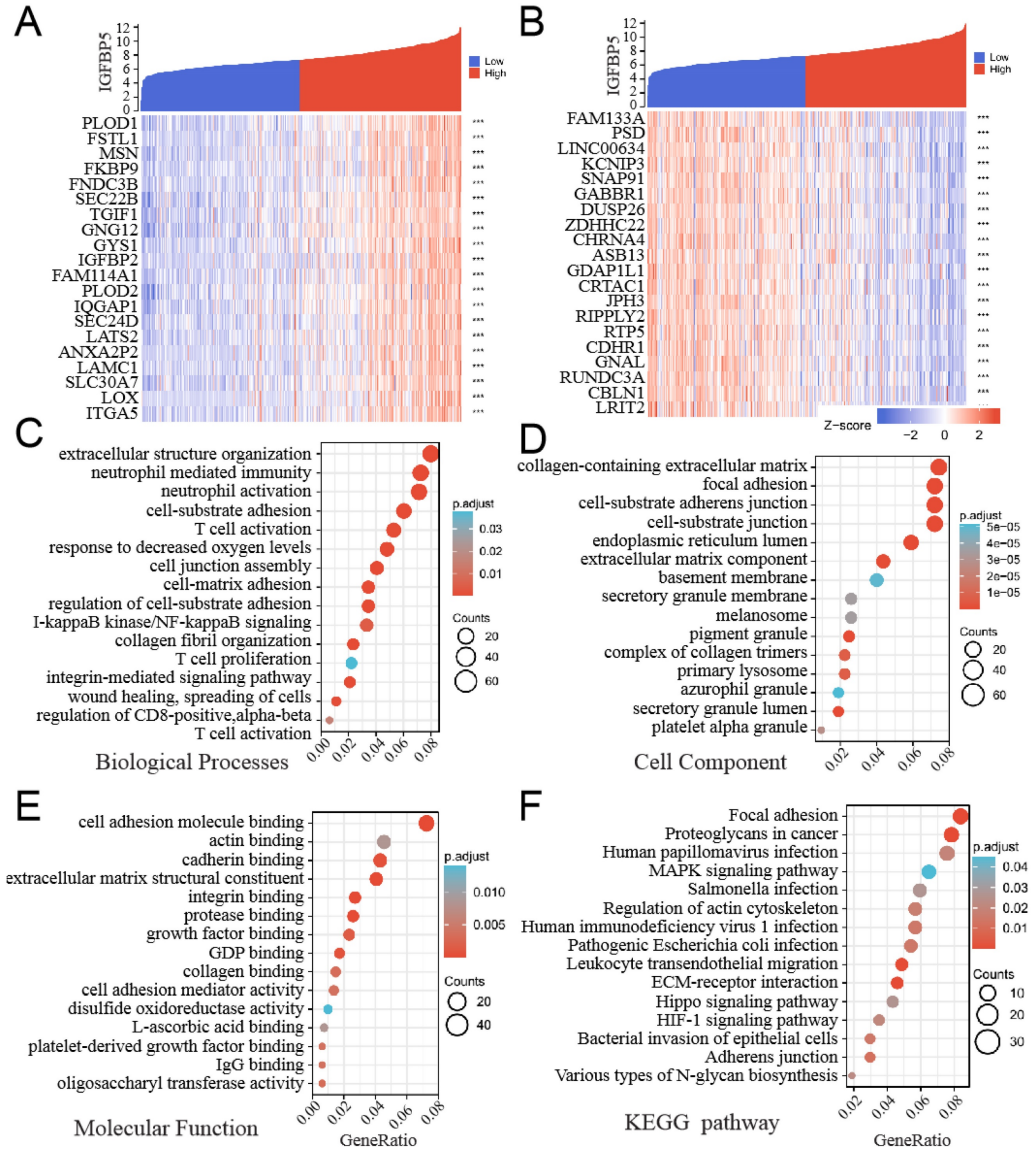
GO and KEGG enrichment analysis of IGFBP5 and its 500 co-expressed genes in glioma patients.** (A, B)** The top 20 genes positively (A) and negatively (B) correlated with IGFBP5 were shown in heatmap in glioma. Data were normalized by Zscore standardization method.** (C-E)** Significant Gene Ontology terms of top 500 genes most positively associated with IGFBP5, including biological processes (C), cell component (D), and molecular function (E). **(F)** Top 15 KEGG enrichment pathways in glioma.

To explore the functions and pathways affected by IGFBP5, we identified genes positively or negatively co-expressed with IGFBP5 using TCGA data in glioma. The top positive and negative 20 genes correlated with IGFBP5 were displayed in a heatmap (Figure 4A, B). We further analyzed potential functional pathways based on the top positive 500 genes using the R software clusterProfiler package. The top 15 significant terms of BP, MF and CC enrichment analysis were presented (Figure 4C-E).

Notably, GO functional of BP enrichment analysis showed that IGFBP5 was associated mostly with extracellular structure organization, neutrophil mediated immunity, cell-substrate adhesion, etc (Figure 4C). In addition, the top 15 KEGG pathways for IGFBP5 and its correlated genes were shown in Figure 6F. Among these pathways, Human immunodeficiency virus 1 infection and Leukocyte transendothelial migration were involved in immunity. Several important pathways were enriched including Focal adhesion, Proteoglycans in cancer, MAPK signaling pathway, HIF-1 signaling pathway, ECM-receptor interaction, Hippo signaling pathway (Figure 4D). These results suggest that high IGFBP5 expression is associated with immunity and multiple oncogenic pathways in glioma.

### Relationship between immune cell infiltration and IGFBP5 expression in glioma

**Figure 5:**
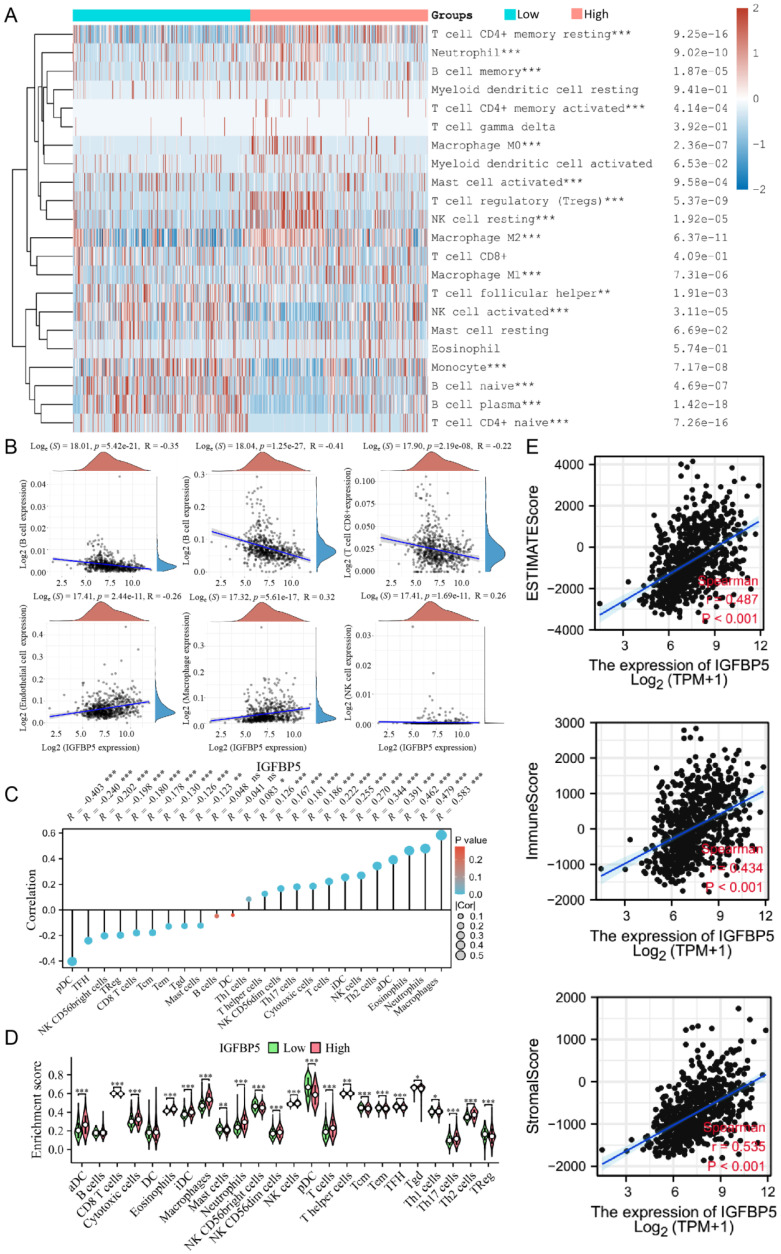
Relationships between IGFBP5 expression and different tumor immune cell infiltration in TCGA database. **(A)** Heatmap revealed the correlation between the expression of IGFBP5 and immune cells using the CIBERSORT algorithm. **(B)** Scatter plot showed the correlation between the expression of IGFBP5 and immune cells by EPIC algorithm. **(C, D)** Spearman's correlation analysis results between IGFBP5 expression and infiltration levels of 24 immune cell types in glioma according to the ssGSEA algorithm. Note: DC, dendritic cells; pDC, plasmacytoid DC; NK, natural killer cells; Tgd, T gamma delta; Th17, type 17 Th cells; iDCs, immature DCs; TReg, regulatory T cells; Tem, T effector memory; Tcm, T central memory; Th1 cells, type 1 Th cells; aDC, activated DC; TFH, T follicular helper; Th2, type 2 Th cells. **(E)** Scatter plot showing the correlation between IGFBP5 expression and immune scores in patients with glioma.

The proportion of 22 types of immune cells in glioma samples retrieved from TCGA database was sorted and analyzed by CIBERSORT algorithm to explore the relationship between IGFBP5 expression and tumor immune microenvironment. The analysis shows that in glioma patients who had high levels of IGFBP5 expression, the levels of T cell CD4+ memory cells, Neutrophil cells, B cell memory cells, Macrophages, Tregs, and NK cell resting cells significantly increased. However, the levels of Monocyte cells, B cell naive, B cell plasma cells, activated NK cells, and T cell CD4+ naive variously decreased (Figure 5A). IGFBP5 expression levels showed negatively correlation with CD8+ T cells, B cells, CD4+ T cells, and positively correlation with Endothelial cells, Macrophages, NK cells based on the EPIC algorithm (Figure 5B). Moreover, the ssGSEA algorithm was performed to analyze the effects of IGFBP5 expression on immune characteristics of 24 types of immune cells. The results showed that IGFBP5 expression positively correlated with the levels of infiltration of aDCs, Cytotoxic cells, Eosinophils, iDCs, NK CD56dim cells, macrophages, neutrophils, NK cells, T cells, T helper cells, Th1 cells, Th17 cells, and Th2 cells (Figure 5C, D). In contrast, CD8 T cells, Master cells, NK CD56bright cells, pDC, Tcm cells, Tem cells, TFH cells, Tgd cells, and TReg cells were decreased in high expression group compared with low expression group (Figure 5C, D). We used the ESTIMATE algorithm to obtain correlations between the expression of IGFBP5 and the immune score (ImmuneScore, StromalScore and ESTIMATEScore) in glioma on data from the TCGA database. The results indicated that IGFBP5 expression was significantly and positively correlated with the ImmuneScore, StromalScore and ESTIMATEScore (all P < 0.01) in glioma (Figure 5E). Next, we analyzed the correlation between IGFBP5 expression and tumor-infiltrating immune cell status based on the expression levels of immune marker gene in glioma using the CGGA and TCGA databases, including B cells, T cells, CD8+ T cells, monocytes, tumor-associating macrophages (TAMs), macrophages, neutrophils, natural killer (NK) cells and dendritic cells. The results showed that IGFBP5 expression was significantly correlated with most biomarkers in different types of immune cells in glioma (**Supplementary Table 3**).

### Correlations between IGFBP5 expression and immunomodulator genes in glioma

**Figure 6:**
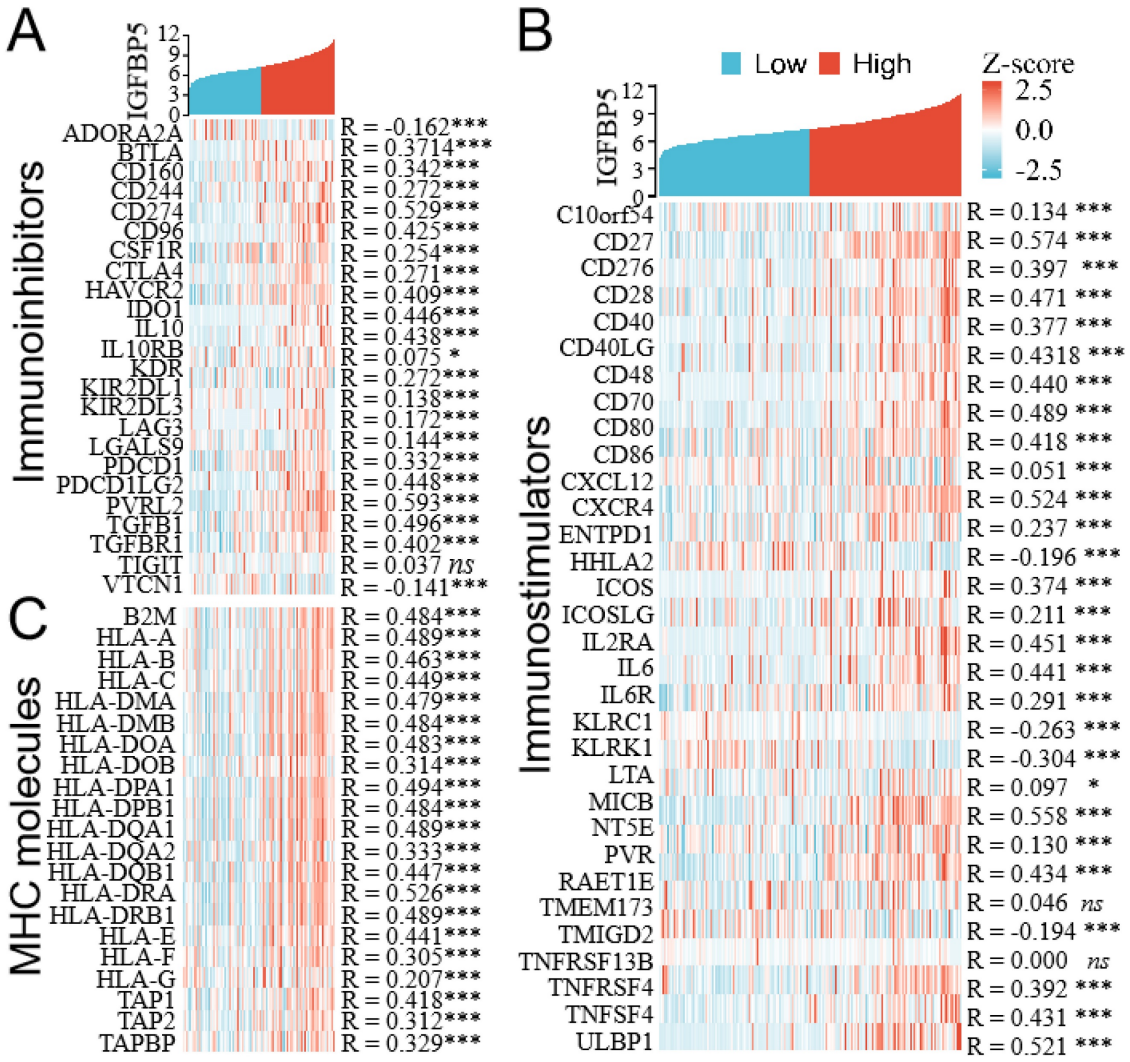
Co-expression of IGFBP5 and immunomodulator genes. **(A)** Heatmap showing co-expression of IGFBP5 and immunoinhibitor genes. **(B)** Heatmap showing co-expression of IGFBP5 and immunostimulator genes. **(C)** Heatmap showing co-expression of IGFBP5 and MHC moleculars.

To evaluate the relationships between IGFBP5 expression and immune-related genes in glioma, we performed an integrative analysis to investigate co-expression of IGFBP5 and immunomodulators (immunoinhibitors, immunostimulators and MHC moleculars). The results revealed that IGFBP5 expression was positively correlated with most immunosuppressive genes (Figure 6A), immunostimulators (Figure 6B), and MHC genes (Figure 6C) in glioma.

Immune checkpoint genes such as PD-L1, PD-1, CTLA4, LAG3, HAVCR2, PDCD1LG2 were positively correlated with IGFBP5 in glioma (Figure 6A). In these immunostimulators genes, CXCR4, CXCL12 belonged to Chemokines and chemokine receptors, were also positively correlated with IGFBP5 expression in glioma (Figure 6B).

### The protein level of IGFBP5 in patients with glioma

**Figure 7:**
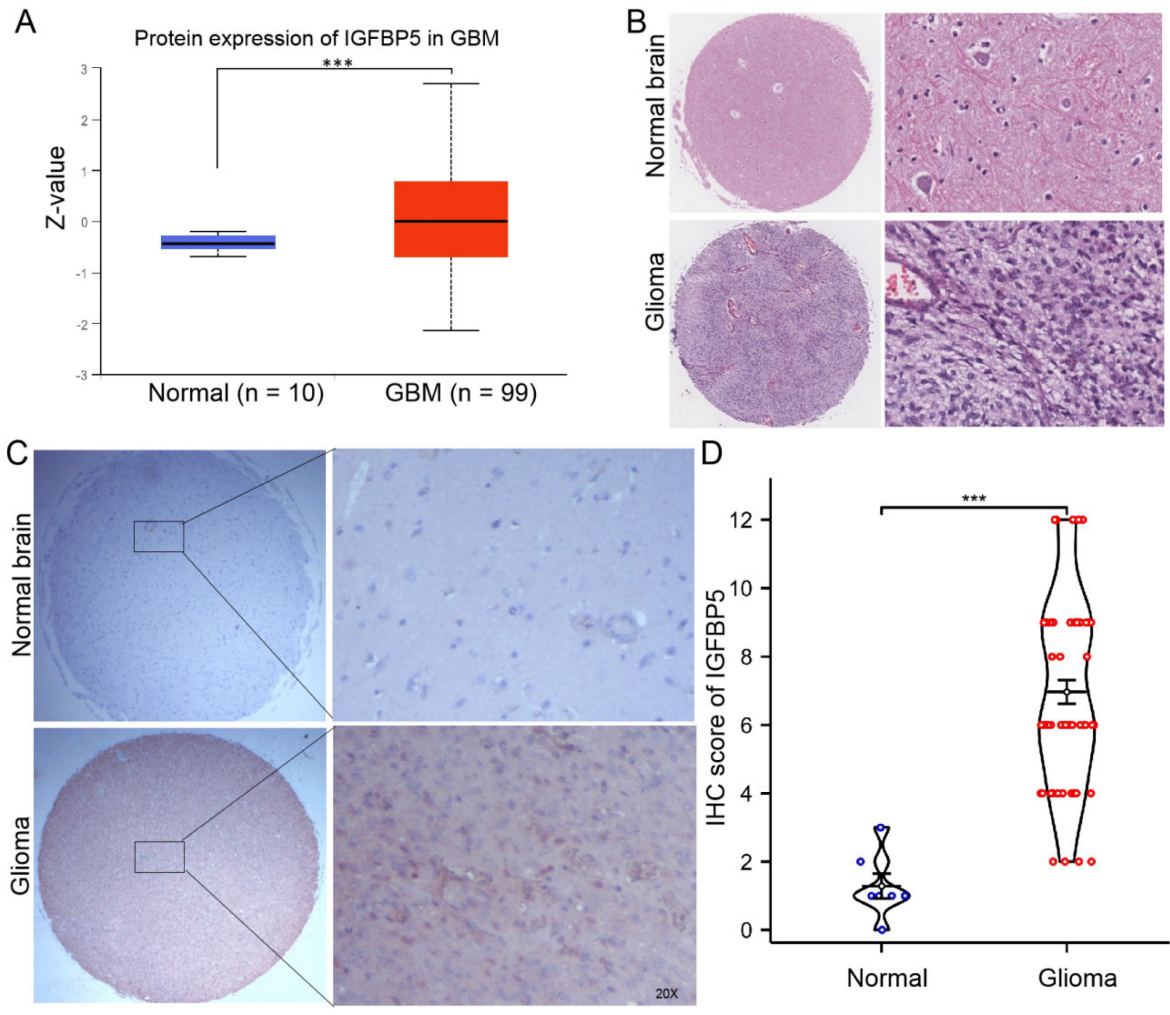
The expression of IGFBP5 in glioma. **(A)** The expression of IGFBP5 in GBM by ULANC. **(B)** Representative images of H&E staining of glioma and normal brain tissues. **(C)** Representative IHC images of IGFBP5 expression in normal brain tissues and glioma tissues. magnification, 200×. D. IHC score of IGFBP5 in clinical tissues, Normal tissue, n = 9; Glioma, n = 76.

To clarify the protein expression of IGFBP5 in glioma, we firstly used the UACLAN database, which is a comprehensive, interactive web resource for analyzing cancer OMICS data. The results found that IGFBP5 was significantly upregulated in GBM compared with normal brain (Figure 7A). H&E staining was performed to show the morphological structure of normal brain and glioma tissues (Figure 7B). Furthermore, immunohistochemical staining of tissue microarray and IHC score were used to analyze the expression of IGFBP5 and revealed that expression of IGFBP5 increased in glioma tissues compared with that in normal brain tissues (Figure 7C, D).

### IGFBP5 promotes cell growth in glioma cells

**Figure 8:**
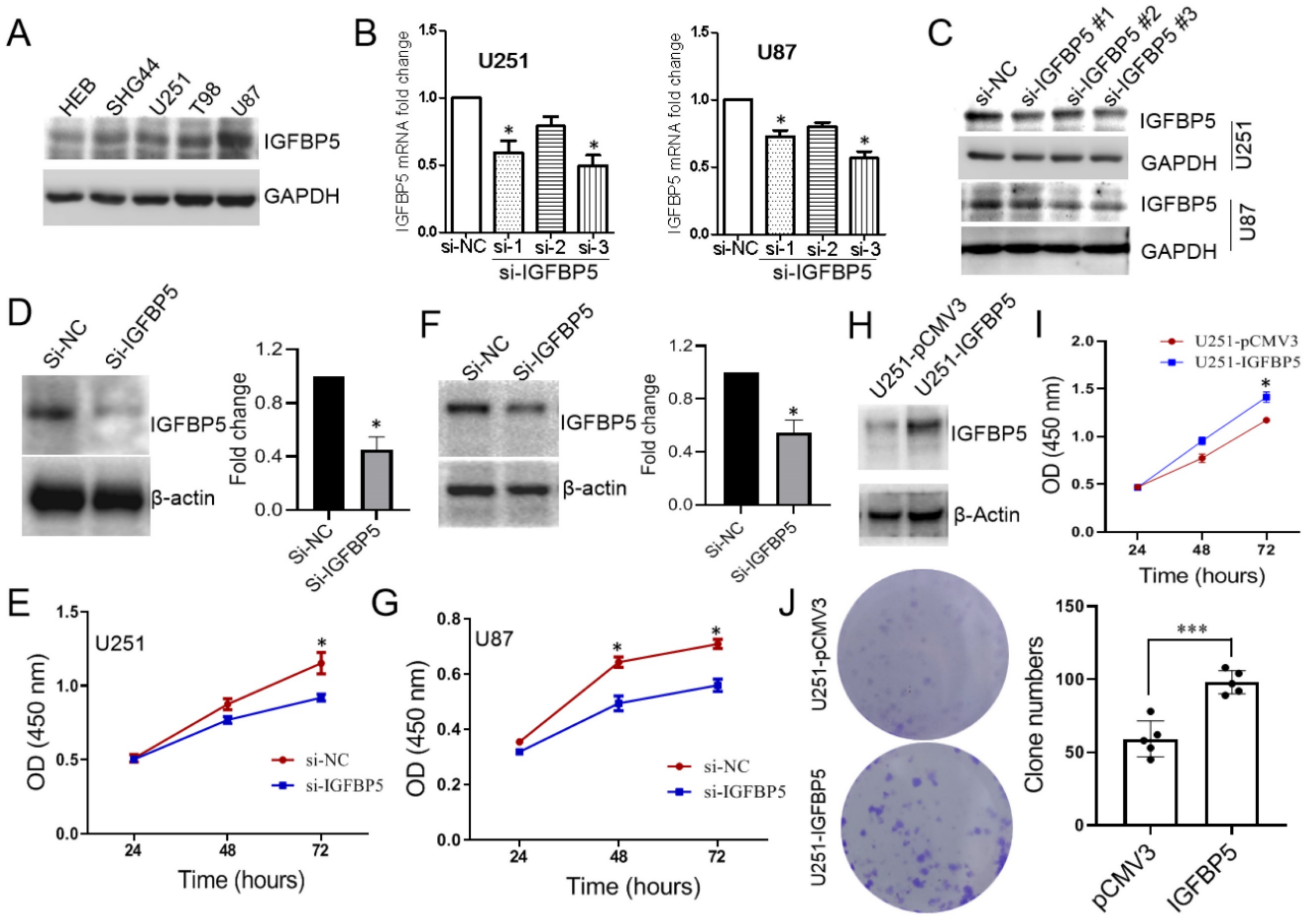
The expression of IGFBP5 was inhibited by IGFP5 siRNA.** (A)** IGFBP5 expressed in astrocyte and glioma cells.** (B)** Knockdown efficiency of IGFBP5 in U251 and U87 cells were tested by qPCR.** (C)** Knockdown efficiency of IGFBP5 in U251 and U87 cells were tested by WB. **(D-G)** IGFBP5 knockdown blocked the proliferation of U251 and U87 cells. **(H, I)** IGFBP5 overexpression increased the proliferation of U251 used CCK-8 assay. **(J)** IGFBP5 overexpression increased the colony formation of U251 cells.

To explore the role of IGFBP5 in glioma cells, we examined the expression levels of IGFBP5 in one human astrocyte cell line, HEB, and four human glioma cell lines, SHG44, U251, U87, T98. The results showed that the expression level of IGFBP5 was the lowest in normal astrocytes (HEB), but significantly higher in glioma cell lines (Figure 8A). We knocked down IGFBP5 expression in U251 and U87 cells via three IGFBP5 siRNAs and qRT-PCR showed that IGFPB5 mRNA expression was significantly reduced transfected with si-TGFBP5 compared to si-NC (Figure 8B). WB also showed that IGFBP5 siRNA could decrease the expression levels of IGFBP5 protein (Figure 8C). The third siRNA for IGFBP5 has the best inhibitor effect and was selected for subsequent research. Then, cck-8 assay was performed and the results showed that inhibition of IGFBP5 could significantly reduce the cell viability of glioma cell lines U251 (Figure 8D, E) and U87 (Figure 8F, G), whereas IGFBP5 overexpression dramatically promoted cell proliferation (Figure 8H, I) and colony formation (Figure 8J) in U251 cell lines. These results indicate that IGFBP5 may act as a tumor oncogene in tumorigenesis of glioma.

### IGFBP5 promotes cell migration and invasion in glioma cells

**Figure 9:**
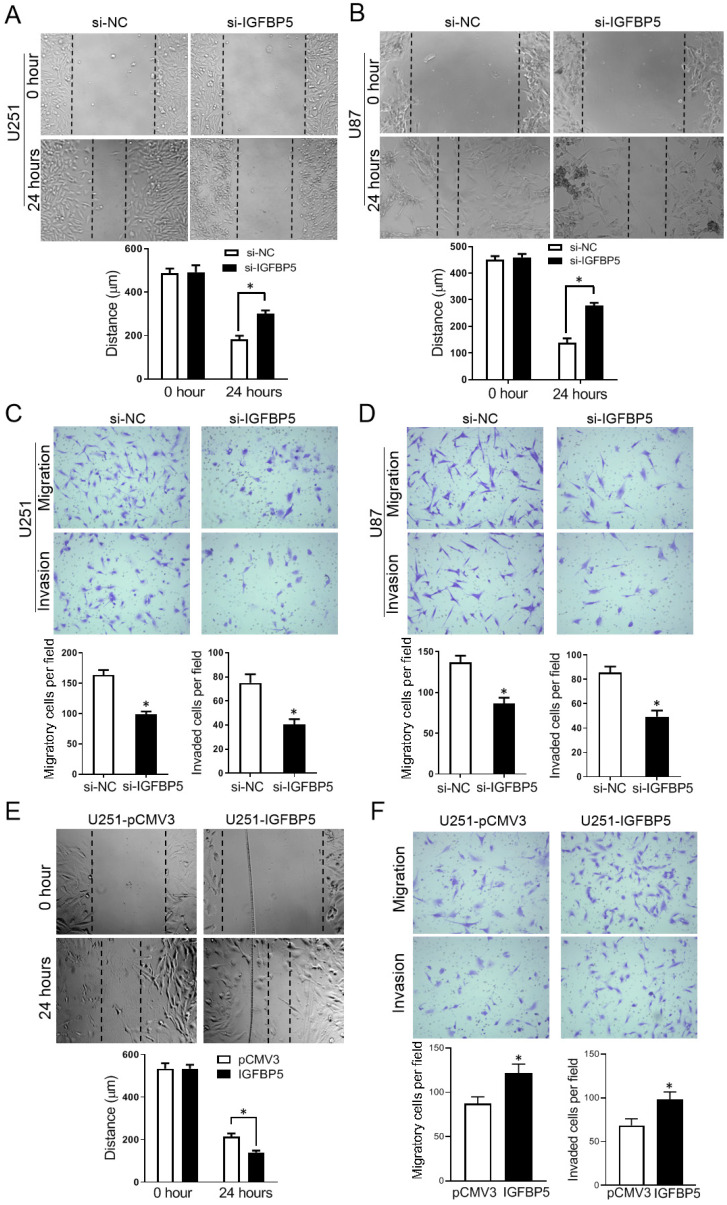
Effect of IGFBP5 on migration and invasion of glioma cells. **(A, B)** IGFBP5 knockdown suppressed the migration of U251 and U87 cells in a scratch wound assay. **(C, D)** IGFBP5 knockdown inhibited the migration and invasion of U251 and U87 cells in a transwell assay. **(E)** IGFBP5 overexpression promoted wound closure used a scratch assay. **(F)** IGFBP5 overexpression the migration and invasion of U251 and U87 cells in a transwell assay.

To explore the function of IGFBP5 on cell migration and invasion, scratch assays and Transwell assays were employed. The scratch assays showed that knockdown of IGFBP5 suppressed the closure of the wound (Figure 9A, B). The transwell assays revealed that IGFBP5 inhibition significantly reduced the number of migrated and invaded cells (Figure 9C, D). On the other hand, when IGFBP5 was overexpressed, the distance of the wound was significantly smaller compared with the control group (Figure 9E). Transwell assays showed overexpression of IGFBP5 elevated the capability of cells crossing the transwell membrane compared with the control group (Figure 9F). Collectively, these data discourse that IGFBP5 expression promotes glioma cell migration and invasion.

### IGFBP5 induces EMT and Hippo-YAP signaling pathway in glioma cells

**Figure 10:**
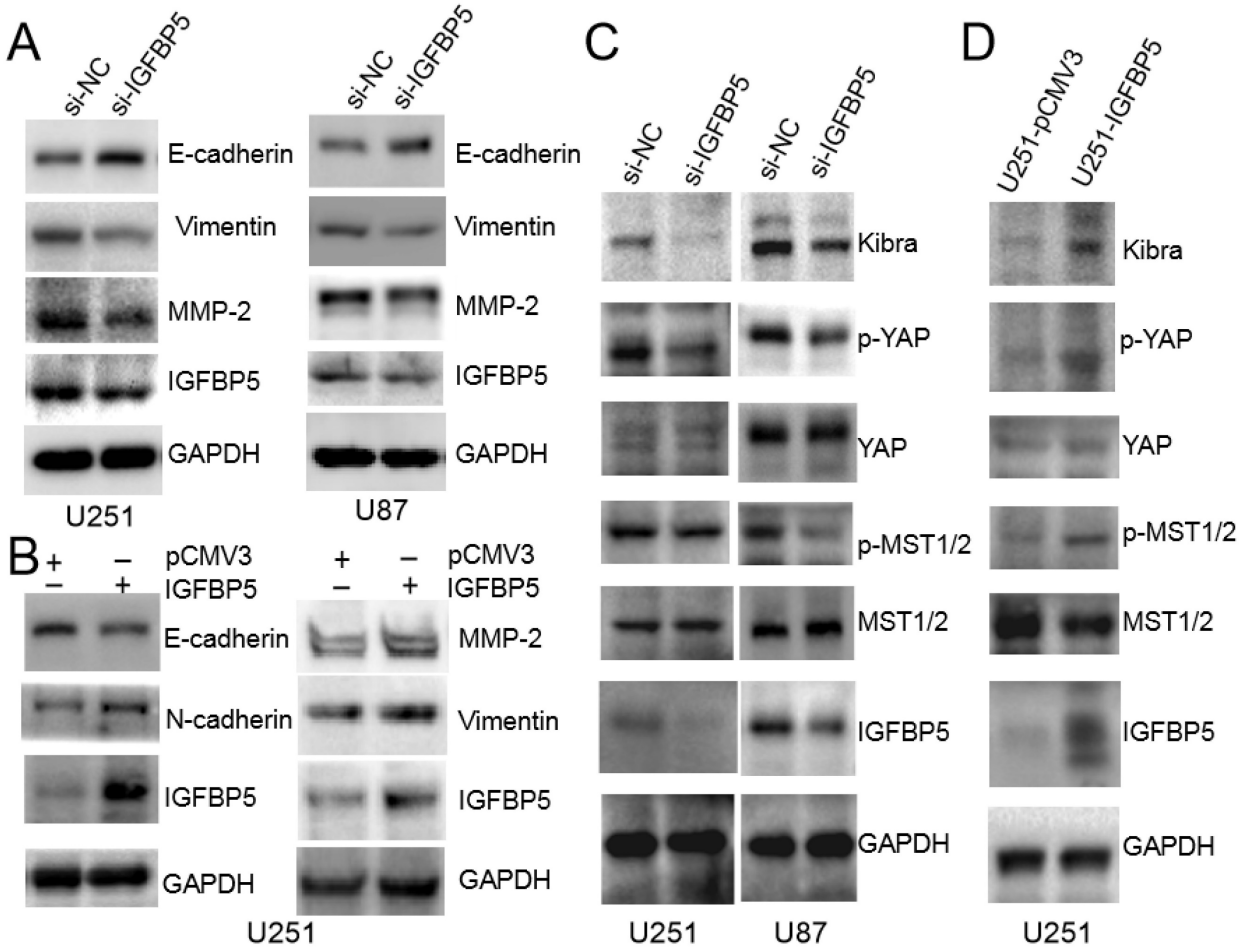
IGFBP5 modulated EMT progress and Hippo-YAP signaling pathway. **(A)**The relative protein levels of E-cadherin,Vimentin and MMP-2 in IGFBP5 knockdown cells compared to corresponding control cells were detected by Western blot. **(B)** The relative protein levels of E-cadherin, N-cadherin, Vimentin and MMP-2 in IGFBP5 over-expression cells compared to corresponding control cells were detected by Western blot. GAPDH was used as a loading control. **(C, D)** Effect of IGFBP5 expression on the expression of proteins related to the Hippo-YAP signaling pathway.

To explore the underlying molecular mechanisms, we examined whether EMT and MMP-2, which are recognized as important factors of glioma invasion 11, 12, were involved in IGFBP5-mediated promotion of glioma cell growth. The protein expression levels of E-cadherin, Viemtnin, N-cadherin and MMP-2, were measured in both IGFBP5 knockdown and IGFBP5- overexpressing cells. Immunoblot analysis confirmed that knockdown of IGFBP5 increased E-cadherin and decreased Vimentin, MMP-2 protein levels (Figure 10A), while overexpression of IGFBP5 decreased E-cadherin protein levels and increased Vimentin, N-cadherin, and MMP-2 protein levels (Figure 10B). Previous bioinformatics analysis showed that IGFBP5 was associated with the Hippo-YAP pathway. Therefore, we used WB to further validate and found that inhibition of IGFBP5 suppressed the YAP signaling pathway, while overexpression of IGFBP5 activated the YAP signaling pathway (Figure 10C, D).

### IGFBP5 regulates PD-L1 and CXCR4 expression in glioma cells

**Figure 11:**
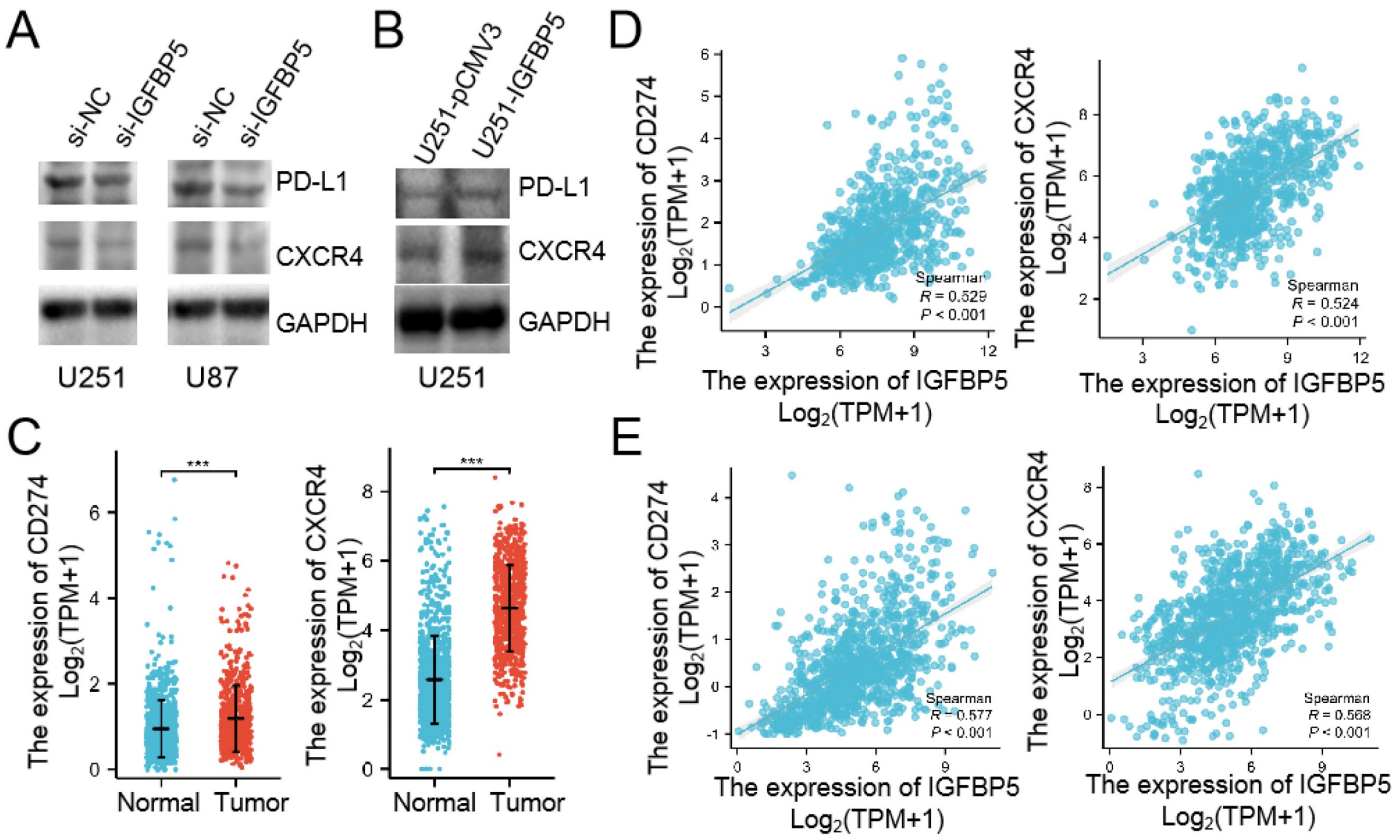
IGFBP5 regulated PD-L1 and CXCR4 expression. **(A)** IGFBP5 knockdown in U251 and U87 cells decreased the expression of PD-L1 and CXCR4. **(B)** IGFBP5 overexpression in U251 cells increased the expression of PD-L1 and CXCR4. **(C)** CD274 and CXCR4 mRNA highly expressed in glioma compared with normal brain based on TCGA database. **(D)** Scatter plot showing the correlation between IGFBP5 expression and and PD-L1, CXCR4 in patients with glioma based on CGGA database.

We already verified that IGFBP5 expression was correlated with immune infiltration in glioma through bioinformatics assay. Then, we used WB to examine whether IGFBP5 inhibition or overexpression could mediated PD-L1 and CXCR4 expression in glioma cells. The results showed that IGFPB5 knockdown reduced PD-L1 and CXCR4 expression in U251 and U87 cells (Figure 11A), while IGFBP5 overexpression increased PD-L1 and CXCR4 expression in U251 cells (Figure 11B). Then, we compared the expression of PD-L1 and CXCR4 mRNA in glioma and normal brain. The results showed that PD-L1 and CXCR4 highly expressed in glioma. Furthermore, we investigated the correlation between IGFBP5 expression and PD-L1, CXCR4 expression in glioma using the CGGA and TCGA databases and the results showed IGFBP5 expression was positively correlated with PD-L1 and CXCR4 expression (Figure 11C, D).

## Discussion

Glioma is the most common form of malignant neoplasm in the central nervous system. To date, the standard treatment for glioma has been dominated by surgery combined with radiation and chemotherapy 13,14. Unfortunately, the prognosis is still poor, particularly for glioblastoma, which shows a 5-year survival rate of 3% 3. It is pivotal to discover promising prognostic molecules and understand the potential mechanisms driving glioma progression.

In the present research, we observed that IGFBP5 was highly expressed in LGG and GBM, and in particular, LGG patients with high expression of IGFBP5 displayed poor prognosis, including OS, PFI and DSS. Importantly, IGFBP5 expression was closely correlated with clinical and prognostic outcomes in glioma using the TCGA, CGGA, Rembrandt, and Gravendeel datasets, including age, tumor grade, IDH mutation status, Histological type, and chromosome 1p/19q co-deletion status, as well as with Primary therapy outcomes. The data also showed that patients with highly-expressed IGFBP5 had worse prognosis than those with lower levels in glioma. Furthermore, the AUC values of 1-, 3-, and 5-year predicted survival rates suggested that IGFBP5 may be a potential factor for predicting poor prognosis in glioma. Cox and univariate analysis revealed that IGFBP5 expression was positively correlated with age, grade, primary therapy outcome, IDH mutation status, and poor OS in glioma patients. According to cox and univariate analysis, a nomogram was constructed to further predict the probability of 1-, 3-, and 5-year OS and the results showed that the sum scores calculated by IGFBP5 expression for each glioma patient could be used to predict OS. Consistent with the bioinformatics results, IHC analysis of tissue microarray demonstrated that IGFBP5 expression was highly expressed in glioma. These results were in accordance with previous studies by Wang et al., who found that IGFBP5 was significantly more expressed in grade IV glioblastoma than in grade II and III glioma 10. Dong et al., also used IHC to reveal that IGFBP5 expression clinically correlated with the progression of glioma, and IGFBP5 was significantly increased in recurrent glioma and correlated with worse survival by the CGGA database 11. Santosh et al., reported the association of IGFBP5 expression with increasing grades of malignancy in astrocytomas 15. Based on our and previous findings, IGFBP5 may be one of the candidate-oncogenes for a diagnosis of poor prognosis in patients with glioma.

In order to discover how IGFBP5 functions biologically, GO and KEGG enrichment analysis were performed. The results revealed that IGFBP5 was positively correlated with different signaling pathways, including I-kappaB kinase/NF-kappB signaling, Proteoglycans in cancer, MAPK signaling pathway, HIF-1 signaling pathway, ECM-receptor interaction, Hippo signaling pathway. Previous studies reported that IGFBP5 modulated pancreatic cancer cell growth and survival through the MAPK or PI3K pathway, and promoted prostate cancer growth via activating the PI3K pathway 16, 17. Overexpression of IGFBP5 affected both canonical and non-canonical signaling pathways, including the IGF-1 signaling, NF-κB signaling, Notch signaling, p38-MAPK signaling, EMT, and PTEN signaling pathways 18. Our results also confirmed that knockdown of IGFBP5 inhibited Hippo-YAP pathway, whereas overexpression of IGFBP5 promoted activation of the Hippo-YAP pathway. IGFBP5 was found to specifically interact with a wide range of proteins, specifically ECM proteins, and was involved in MMP-mediated migration and invasion 19, 20. However, the function of IGFBP5 in tumors was controversial. IGFBP5 expression promoted pancreatic cancer cell growth 21. And IGFBP5 could attenuate the inhibitory effect of miR-204-5p on growth in papillary thyroid carcinoma 22. In contrast, IGFBP5 could suppress the growth or proliferation in gastric cancer cell and breast cancer 23, 24. In glioma, a previous study revealed that IGFBP5 promoted cell invasion involved in EMT, but inhibited cell proliferation 11. In this study, we found that IGFBP5 knockdown significantly inhibited glioma cell proliferation, migration and invasion, while IGFBP5 overexpression promoted glioma cell proliferation, migration and invasion Knockdown IGFBP5 expression inhibited EMT and MMP-2 expression, while increasing IGFBP5 expression promoted EMT and MMP-2 expression, which indicated that IGFBP5 facilitated glioma cell migration and invasion involved in EMT and MMP expression, which is consistent with the part results of the prevoius study 11. However, the exact molecular mechanisms of IGFBP5 involved in EMT remain unclear.

Evidence showed that immune infiltration and tumor microenvironment (TME) played a key role in tumor development, therapeutic response, and clinical outcome 25, 26. Glioma was a group of immunosuppressive tumors with a complex TME, which may be one of the main reasons limiting the efficacy of immunotherapy 27, 28. The functions of IGFBP5 in the TME have not been reported in glioma. Therefore, CIBERSORT, EPIC, quanTiseq and ssGSEA were used to investigate the correlation between IGFBP5 expression and infiltration of immune cells in glioma. Analysis of immune cell infiltration in the present study revealed that IGFBP5 expression was correlated with the infiltration level of immune cells, including TAM and M2 macrophage, CD8+ T cells, CD4+ T cells, NK cells, and B cells. Macrophages were one of the most important immune cells and account for 30-50% of the glioma TME and modified the immune microenvironment of the tumor by adjusting levels of angiogenic and immunosuppressive genes 29-31. Clinically, Tumor-associated macrophages (TAMs) in brain were associated with poor outcomes and their accumulation was associated with elevated tumor grade. Furthermore, TAMs promoted numerous tumor-promoting activities, enhanced cellular invasion and immune suppression 32, 33. Previous reports have shown that the proliferation of glioma cells is accelerated by macrophages and may account for the higher cancer incidence and poor prognosis in patients with high-grade glioma 34, 35.

Thus, it may be an appealing approach to enhance the survival of pateints with glioma via targeting the infiltrated TAMs. Our results showed that IGFBP5 expression was significantly and positively correlated with levels of macrophages in glioma. All these results suggest that IGFBP5 may promote tumor metastasis via recruiting infiltration of macrophages and leading to development of immune-suppressive tumor environment. In brain tumors, NK cells exerted the tumor-suppressing function both *in vitro* 36 and *in vivo* 37. B cell can act as antigen-presenting cells (APC), which may be a crucial factor for anti-tumor immunity and tumor regression via T cells in a GBM model 38. Our results indicated that higher expression of IGFBP5 was associated with a reduction of B and NK cells in glioma, and that less either B or NK cells could activate antitumor immune response and induce immune escape, which in turn, contributed to poor prognosis. Go analysis alsoshowed that IGFBP5 expression was significantly involved in T cell activation, T cell proliferation, and regulation of CD8+ T-cell activation. Our correlation analysis showed that IGFBP5 was associated with CD4+ T, CD8+ T cells by different algorithms. Some inconsistencies of the results can be due to inconsistent data processing by different algorithms. We further found that a strong link between IGFBP5 expression and immunosuppressive genes, MHC genes, immunostimulator genes in glioma. Importantly, there was a positive correlation between IGFBP5 expression and immunosuppressive genes such as TGFB1, PD-L1, and IL10 further demonstrating that IGFBP5 is critical in the regulation of tumor immunoregulation, and the polarization of macrophages 39, 40. Specifically, we found that, as IGFBP5 levels increased, the expression of the seven immune checkpoint genes (BTLA, CD274, CTLA4, HAVCR2, LAG3, PDCD1, and PDCD1LG2) were increased and a large positive correlation was observed between IGFBP5 and these immune checkpoints in glioma. The highly-expressed PD-L1 on tumors was demonstrated to induce anti-tumor immune inhibition by promoting the apoptosis of antigen-specific and tumor-reactive T cells 41 and the PD-1/PD-L1 axis could enhance invasion of GBM cells in brain 42. Especially, *in vitro* assays indicate that IGFBP5 regulates the expression of PD-L1 in glioma. Targeting immune checkpoints, which elevated anti-tumor immune responses, has led to a major clinical advance, and provides novel targets for tumor treatment 43, 44. The analysis showed that strong correlations were shown between IGFBP5 and immune checkpoints, suggesting that IGFBP5 could serve as a immunotherapeutic target for glioma. Despite these fndings, there are several limitations. The present study assesses the expression and biological roles of IGFBP5 in databases of patients with glioma and cultured cells, not *in vivo*. As we know, each type of immune cell functions is different, even conversely in the tumor microenvironment. Therefore, the exact function and detailed mechanism of IGFBP5 in regulating the cell invasion and tumor microenvironment in glioma is required for further research.

In summary, the findings of this study show that IGFBP5 expression is increased in glioma tissues and that its high expression correlates with malignant progression of glioma. In the glioma cells, knockdown of IGFBP5 leads to less proliferating, migrating and invading activities, while overexpression has the opposite effect. Mechanistically, we uncover that IGFBP5 promotes cell migration and invasion by regulating EMT and Hippo-YAP signaling pathway. Moreover, IGFBP5 may modulate the infiltration of immune cells in glioma microenvironments. Overall, our findings suggest that IGFBP5 is strongly involved in glioma development, and may serve as a potential prognostic biomarker and immunotherapeutic target for patients with glioma.

## Supplementary Material

Supplementary figures and tables.

## Figures and Tables

**Table 1:** Relationship between IGFBP5 expression and clinicopathological features in the glioma.

Characteristics	Low expression of IGFBP5	High expression of IGFBP5	P value
n	349	350	
**Age, n (%)**			< 0.001
<= 60	315 (45.1%)	241 (34.5%)	
> 60	34 (4.9%)	109 (15.6%)	
**Gender, n (%)**			0.062
Female	161 (23%)	137 (19.6%)	
Male	188 (26.9%)	213 (30.5%)	
**WHO grade, n (%)**			< 0.001
II	161 (25.3%)	63 (9.9%)	
III	129 (20.3%)	116 (18.2%)	
IV	16 (2.5%)	152 (23.9%)	
**IDH status, n (%)**			< 0.001
WT	36 (5.2%)	210 (30.5%)	
Mut	312 (45.3%)	131 (19%)	
**Primary therapy outcome, n (%)**			< 0.001
PD	47 (10.1%)	65 (14%)	
SD	96 (20.6%)	52 (11.2%)	
PR	42 (9%)	23 (4.9%)	
CR	97 (20.9%)	43 (9.2%)	
**1p/19q codeletion, n (%)**			< 0.001
Non-codel	194 (28%)	326 (47.1%)	
Codel	155 (22.4%)	17 (2.5%)	
