# Supplementary material for: IGFBP5, as a Prognostic Indicator Promotes Tumor Progression and Correlates with Immune Microenvironment in Glioma
Source: J Cancer. 2024 Jan 1;15(1):232–50. doi: 10.7150/jca.87733 (PMC10751672; doi:10.7150/jca.87733)
Supplement: Supplementary file 1 — Supplementary figures and tables. [file jcav15p0232s1.pdf]

Supplementary Figures and Tables

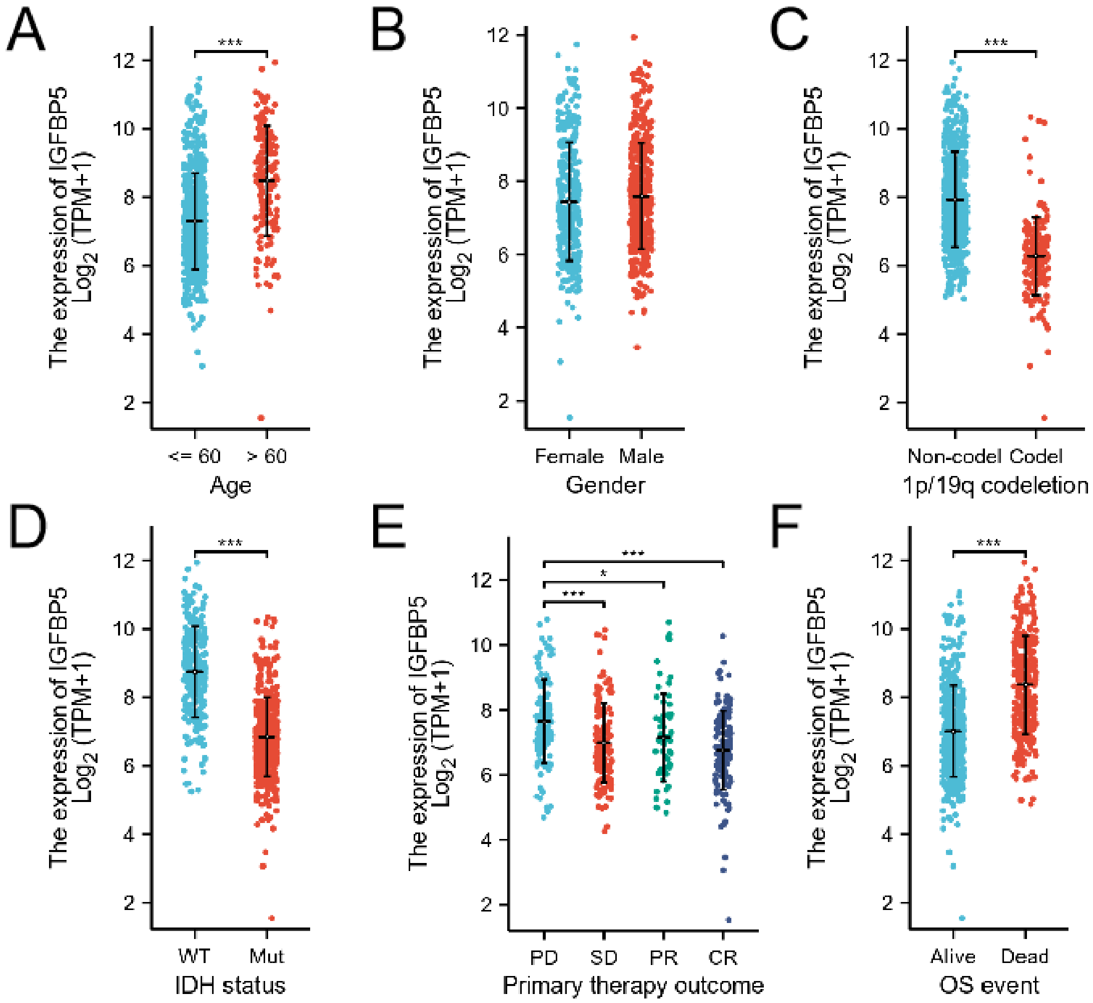

Supplementary Figure 1. The correlation between IGFBP5 expression and different Clinical Parameters. (**A-F**) The correlation between IGFBP5 expression and different clinical features, including the age (A), gender (B), 1p/19q codeletion (C), IDH mutation status (D), primary therapy outcome (E) and Overall Survival (F). Primary therapy outcome: including PD, progressive disease; SD, stable disease. PR, partial response; CR, complete response.

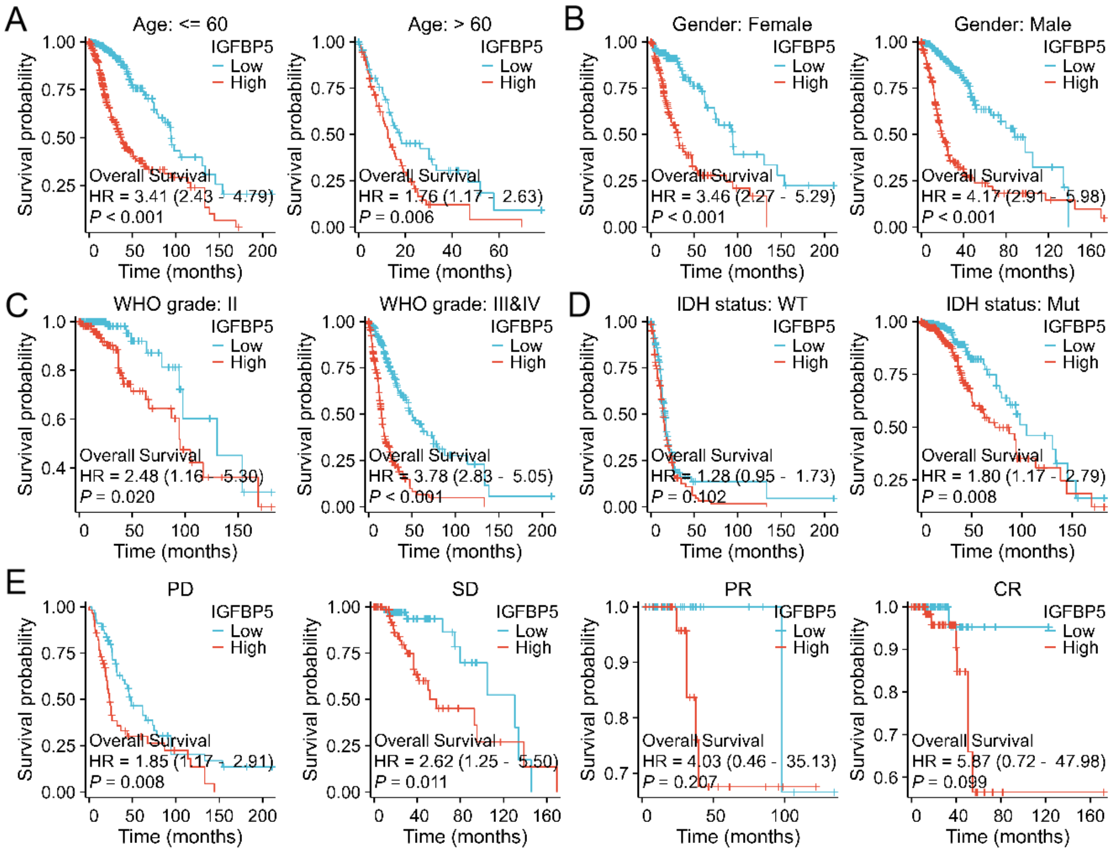

Supplementary Figure 2. Analysis of the prognostic value of IGFBP5 in different glioma subgroups. Analysis of the prognostic value of IGFBP5 in different subgroups, including (**A**) age, (**B**) gender, (**C**) WHO grade, (**D**) IDH mutation status, and (**E**) Primary therapy outcome. PD: progressive disease; SD: stable disease; PR: partial response; CR: Complete response.

Supplementary Table 1 Four Datasets used in this study

Dataset	Data type	WHO grade II	WHO grade III	WHO grade IV
TCGA	RNA-seq	224	245	168
CGGA	RNA-seq	291	334	388
Rembrandt	Microarray	98	85	130
Gravendeel	Microarray	24	85	159

Supplementary Table 2 The primers and siRNAs of IGFBP5

Primers	IGFBP5	5'-ACAAGAGAAAGCAGTGCAAACC-3'
		5'-CGTCAACGTACTCCATGCCT-3'
	GAPDH	5'-GCGTGACATTAAGGAGAAGC-3'
		5'-CCACGTCACACTTCATGATGG-3'
si-RNAs	si-NC	5'-UUC UCC GAA CGU GUC ACG UTT-3'
		5'-ACG UGA CAC GUU CGG AGA ATT-3'
	si-IGFBP5 (＃1)	5'-GCU GAC CCA GUC CAA GUU UTT-3'
		5'-AAA CUU GGA CUG GGU CAG CTT-3'
	si-IGFBP5 (＃2)	5'-UCA UCU CUG CAC CUG AGA UTT-3'
		5'-AUC UCA GGU GCA GAG AUG ATT-3'
	si-IGFBP5 (＃3)	5'-GUG ACC GCA AAG GAU UCU ATT-3'
		5'-UAG AAU CCU UUG CGG UCA CTT -3'

Supplementary Table 3 The correlation of IGFBP5 expression with immune markers of glioma

Description	Gene marker	CGGA		TCGA	
		Cor	p	Cor	p
CD8+ T cell	CD8A	0.37	***	0.36	***
	CD8B	0.25	***	0.38	***
T cell (general)	CD3D	0.15	***	0.37	***
	CD3E	0.35	***	0.38	***
	CD2	0.35	***	0.41	***
B cell	CD19	-0.02	ns	0.17	***
	CD79A	0.08	**	0.21	***
	CD27	0.05	ns	0.20	***
Monocyte	CD14	0.35	***	0.39	***
	CSF1R	0.37	***	0.24	***
TAM	CCL2	0.52	***	0.45	***
	CD68	0.51	***	0.43	***
	IL10	0.42	***	0.37	***
M1 Macrophage	NOS2	0.30	***	0.35	***
	IRF5	0.34	***	0.31	***
	IL6	0.45	***	0.36	***
M2 Macrophage	CD163	0.55	***	0.55	***
	VSIG4	0.47	***	0.44	***
	MS4A4A	0.48	***	0.42	***
Dendritic cell	ITGAX	0.33	***	0.25	***
	CD1C	0.27	***	0.49	***
	NRP1	0.66	***	0.50	***
	THBD	060	***	0.48	***
Neutrophils	CCR7	0.35	***	0.33	***
	ITGAM	0.41	***	0.38	***
Natural killer cell	KIR2DL1	/	/	0.13	***
	KIR2DL3	/	/	0.13	***
	KIR3DL1	/	/	0.13	***
	KIR3DL3	/	/	0.04	ns
Th1	STAT4	-0.02	ns	-0.09	*
	TBX21	0.12	***	0.23	***
	CD4	0.55	***	0.39	***
Th2	GATA3	0.32	***	0.39	***
	CCR4	0.24	***	0.36	***
	CCR8	/	/	0.22	***
Treg	FOXP3	0.11	**	0.07	ns
	STAT5B	0.45	**	-0.09	*
	TGFB1	0.35	***	0.48	***
T cell exhaustion	CD274	0.58	***	0.53	***
	CTLA4	0.21	***	0.26	***
	HAVCR2	0.48	***	0.39	***

TAM, tumor-associated macrophage; Th, T helper cell; Tfh, Follicular helper T cell; Treg, regulatory T cell; Cor, R value of Pearson's correlation; ns, no significant difference. $^{*}P < 0.05$; $^{**}P < 0.01$; $^{***}P < 0.001$.
